# Magnetic Resonance-Guided Diagnosis of Spontaneous Intracranial Hypotension in a Middle-Aged Woman

**DOI:** 10.1155/2022/4438923

**Published:** 2022-02-21

**Authors:** Jordan Hughes, Briana Chavez

**Affiliations:** ^1^Mountainview Regional Medical Center, Las Cruces, NM, USA; ^2^Burrell College of Osteopathic Medicine, Las Cruces, NM, USA

## Abstract

Spontaneous intracranial hypotension (SIH) is a rare condition caused by a *cerebrospinal* fluid (CSF) leak. It is diagnosed by clinical features that include an orthostatic headache combined with imaging findings demonstrating intracranial hypotension and a CSF leak. We present the case of a 45-year-old woman with an orthostatic headache who was found to have a sagging brain with a downward-displaced cerebellum and pachymeningeal enhancement with gadolinium contrast. This was initially misidentified as a Chiari I malformation, but the constellation of symptoms and MRI findings were later recognized as characteristic of SIH. Diagnosis of SIH and a CSF leak was confirmed with CT myelography. She was treated with a nontarget epidural blood patch, and her symptoms resolved. An orthostatic headache, a sagging brain, and pachymeningeal enhancement on MRI are highly specific for SIH, raising suspicion for this uncommon and often missed diagnosis.

## 1. Introduction

Spontaneous intracranial hypotension (SIH) is an uncommon condition characterized by a low volume of CSF. The most common presenting symptom is an orthostatic headache, although rare symptoms can include cranial nerve palsies, behavior changes, reversible dementia, and movement disorders [1–5]. The estimated incidence is 5 in 100,000, with a female to male predominance of 2 : 1 and a peak incidence of 40 years of age [6]. Pathophysiology involves a CSF leak with proposed predisposing factors including connective tissue disorders, spinal meningeal diverticulum, and spinal osteophytes [7–9]. Although in most cases, the inciting factor leading to a CSF leak is unknown, there are potential events such as strenuous activity, sneezing, and coughing that could have produced a dural meningeal tear in some cases [10]. Diagnosis often relies on radiological evidence of a CSF leak, including MRI and CT myelography or a lumbar puncture demonstrating low opening pressure (<60 mmHg) [11]. Misdiagnosis of SIH is common, and delayed treatment can often lead to serious or even fatal complications that include cerebral hemorrhage, sinus venous thrombosis, subdural hematoma, uncal or tonsillar herniation, and brainstem ischemia [12]. It is therefore important to recognize the clinical presentation and distinguishing features that can lead to a diagnosis of SIH for prompt treatment. We present a case with both atypical and typical features that was initially misidentified. We discuss the features that distinguish this often missed and uncommon diagnosis from other diagnoses.

## 2. Case Presentation

**Figure 1:**
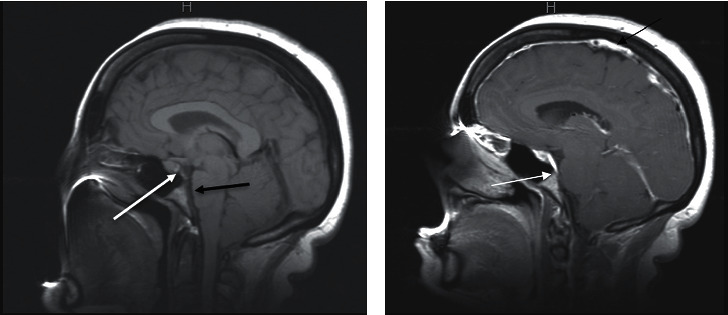
(a) Sagittal T1-weighted MRI image demonstrating the sagging brain and downward displacement of the cerebellum, pituitary enlargement, closure of the midbrain-pons angle (white arrow), and flattening of the pons (black arrow). (b) Postcontrast sagittal T1 image demonstrating diffuse pachymeningeal enhancement, engorgement of dural venous sinuses (black arrow), and obliteration of the prepontine cistern (white arrow).

**Figure 2:**
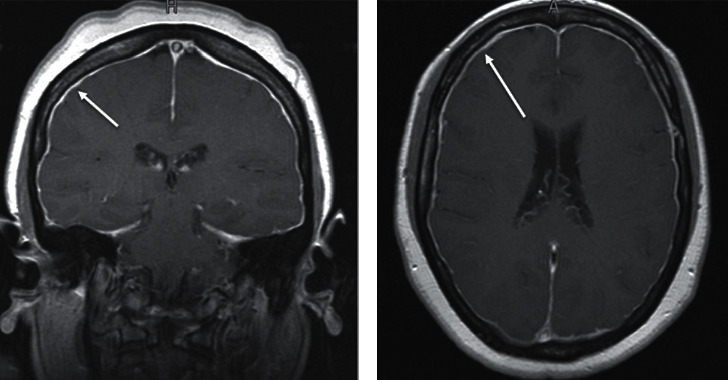
Coronal (a) and axial (b) T1W MRI images with gadolinium enhancement, demonstrating diffuse pachymeningeal enhancement (arrows).

**Figure 3:**
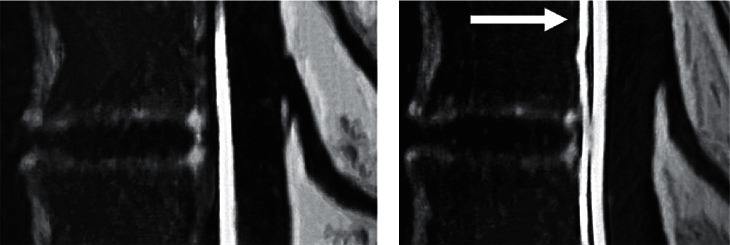
Sagittal view of CT myelography of thoracic spine T10-T11 at 10 seconds (a) and 20 seconds (b) revealing a paravertebral collection indicating a CFS leak (white arrow).

A 45-year-old Hispanic woman with a history of hypertension and chronic migraines presented to a neurology clinic with a worsening headache, dizziness, tinnitus, and gait imbalance. She was also experiencing associated nausea, vomiting, and photophobia. The only things that improved her symptoms were lying supine, on her side, or taking over-the-counter pain medications containing caffeine. Her symptoms began two months prior to presentation and had gradually worsened over that period. At the time of presentation, she was afebrile and reported no recent illness or sick contacts. She reported no recent trauma. No focal neurological deficits were noted on her physical exam. A brain MRI with and without contrast was performed due to the acute worsening of her symptoms. MRI demonstrated sagging of the brain (Figure 1) and pachymeningeal contrast enhancement with a subdural fluid collection (Figure 2). Her condition was initially misidentified as a Chiari I malformation; however, she was eventually referred to neurosurgery where CT myelography demonstrated a CSF leak in the thoracic spine levels T10-T11 (Figure 3). The CSF pressure was too low to be measured. A single nontargeted epidural blood patch was performed without complications. The patient reported improvement in her symptoms and was discharged from the hospital the following day. Upon one-month follow-up to the neurology clinic, the patient no longer experienced headaches or dizziness and was without any other symptoms or focal neurological deficits.

## 3. Discussion

As previously mentioned, this case was initially misdiagnosed as a Chiari I malformation. This can be common due to the overlap of clinical presentations and similarity that can be seen upon imaging. SIH has even been described as a “pseudo-Chiari I malformation” due to the striking similarities [13]. The pathogenesis of SIH and Chiari I, however, is different and requires distinct clinical approaches and treatments. It is therefore important to distinguish the two in order to provide the best patient care and outcomes.

Diagnosis of SIH is based on a clinical presentation including an orthostatic headache and radiological evidence of a CSF leak, usually by an MRI or CT myelography [11]. Recent advances in MRI technology have greatly enhanced the ability to accurately diagnose many neurological pathologies, including SIH. As seen in this case, an MRI helped identify displacement of the cerebellum (Figure 1). Chiari malformation is a more common disorder that similarly presents with downward displacement of the cerebellum and can often lead to misdiagnosis. However, the underlying mechanism for the downward-displaced cerebellum in SIH is caused by reduced buoyancy and “sagging” of the brain due to CSF volume [14]. In contrast, Chiari I is a congenital malformation hypothesized to be caused by underdevelopment of the bony structures of the posterior fossa, causing crowding within the cranium [15]. Also, there are no subdural effusions and no pachymeningeal enhancement in Chiari I. In addition, MRI may show pachymeningeal gadolinium enhancement and engorgement of the dural venous sinuses on T1- weighted images in patients with SIH [16] (Figure 2). This is caused by enlargement of the dural arteries or dilatation of cortical and medullary veins and dural venous sinuses [17]. This finding can be helpful in distinguishing SIH from other neurological abnormalities such as Chiari I malformation.

Additional MRI findings associated with SIH are subdural effusions, closure of the midbrain-pons angle, enlargement of the pituitary, flattening of the pons, and obliteration of the prepontine cistern [16, 17] (Figure 1). These findings can be used to further help distinguish SIH from similar-appearing pathologies. These signs may also be associated with the degree of downward brain descent [18]. Closure of the midbrain-pons angle may be associated with a poorer response rate to the first attempted epidural blood patch [19]. Therefore, MRI brain imaging not only has diagnostic value in SIH but also has the potential to be predictive of the degree of the disease and the potential for treatment response.

CT myelography is often considered the gold standard for diagnosis, as it offers direct visualization of the spinal canal and potential CSF leaks. This involves intrathecal contrast injection and visualization of the spinal canal. This can offer further insight and can often localize tears in the dura or arachnoid. Diagnosis in this case was confirmed using this method (Figure 3). Lumbar puncture can also be used in diagnosis, although it may have low or unobtainable opening pressure, making this less useful for diagnostic purposes unless diagnosis is uncertain. CT cisternography is used similarly to myelography and requires intrathecal contrast injection. The patient is, however, tilted head down to better visualize CSF leaks secondary to fractures in the base of the skull.

Patients have been found to have improvement with hydration and caffeine, but an epidural blood patch of leak is often warranted. This procedure involves a blood draw of 20 mL or greater that is then injected into the epidural space [19]. This is often performed in a nontargeted approach in the lumbar spine. This is thought to tamponade the CSF leak in the short term and cause fibrin deposition and scar tissue formation in the long term [20]. Occasionally, a targeted blood patch is performed when the site of the CSF leak can be localized. A single epidural blood patch is often curative, as was seen in this case, although refractory and complicated cases may require multiple blood patches or neurosurgical intervention.

## 4. Conclusions

SIH is a rare and often missed diagnosis that requires careful examination of radiological signs for accurate diagnosis and distinction from differential diagnosis. The presented case highlights a constellation of MRI findings demonstrating brain sagging unique to SIH that includes downward cerebellar displacement, enlarged pituitary, closure of the midbrain-pons angle, and flattening of the pons. These findings, in combination with pachymeningeal gadolinium contrast enhancement, are specific to SIH and can provide an accurate diagnosis to guide proper management.
